# The role of autophagy in calcium oxalate kidney stone: A systematic review of the literature 

**DOI:** 10.3389/fphys.2022.1008264

**Published:** 2022-09-21

**Authors:** Hao Li, Yingjian Zhou, Wenchao Xu, Jihong Liu, Shaogang Wang, Hongyang Jiang

**Affiliations:** ^1^ Department of Urology, Tongji Hospital, Tongji Medical College, Huazhong University of Science and Technology, Wuhan, China; ^2^ Institute of Urology, Tongji Hospital, Tongji Medical College, Huazhong University of Science and Technology, Wuhan, China

**Keywords:** autophagy, calcium oxalate stone, kidney stone, nephrolithaisis, calcium oxalate

## Abstract

**Background:** Calcium oxalate kidney stone is one of the common diseases in the urinary system and has a high recurrence rate. Currently, the pathogenesis of kidney stone and the methods to prevent recurrence are still being investigated. Autophagy, as an event of cellular self-repair, has received attention in the field of kidney stone in recent years. In some current studies, autophagy has shown destructiveness and protectiveness in the pathogenesis of kidney stone. The inhibition or promotion of autophagy may be a key target for future kidney stone therapy. This systematic literature review discusses the function of autophagy in kidney stone pathogenesis in the context of current research and synthesizes the evidence analysis to provide a basis for new future therapies.

**Method:** We systematically reviewed the literature during September 2021 according to the Preferred Reporting Items for Systematic Evaluation and Meta-Analysis (PRISMA) guidelines. Articles on studying the role of autophagy in the pathogenesis of calcium oxalate kidney stone were extracted from PubMed, MEDLINE, Embase and Scopus, including *in vivo* versus *in vitro* experiments. The study topic, language and publication date were not restricted. Two authors (Li and Zhou) searched and screened the literature.

**Results:** We screened 18 articles from the 33 collected articles, of which 6 conducted *in vitro* cellular studies, four conducted animal studies, eight conducted cellular studies with animal studies, and five studied human specimens. In early studies, the literature generally concluded that autophagy is deleterious in the development of kidney stone. In 2020, the idea of the protectiveness of autophagy associated with kidney stone was first proposed and focused on targeting transcription factor EB. In addition, the interaction of autophagy with other cellular events and the regulation of signaling molecules are focused on in this paper.

**Conclusion:** This systematic review provides advances in research on the role of autophagy in renal calculi. The current studies suggest that both upregulation and downregulation of autophagy may ameliorate injury in kidney stone models. The authors prefer the upregulation of autophagy as a future research direction for kidney stone treatment.

## Introduction

Kidney stone is one of the most common diseases in urology, with a recurrence rate of up to 50%, and the majority of kidney stone cases are calcium oxalate stones (Kusmartsev et al., 2016; Zeng et al., 2017). Its prevalence has increased over the past few decades. Kidney stone is a disease caused by a combination of factors, including genes, environment, metabolism, etc. (Ziemba and Matlaga, 2018). The high morbidity and recurrence rates of kidney stone make it a burden on the medical and healthcare system and exacerbate the pain of secondary treatment for patients (Hiatt et al., 1982). A large number of studies have been conducted to address the issue of kidney stone pathogenesis, but no definitive explanation has been obtained and no significant contribution has been made to the actual treatment of kidney stone and prevention of recurrence. The treatment of kidney stone is currently mainly surgical. Although minimally invasive surgery and robotic surgery have advanced rapidly in recent years, no clearly effective drugs have been found for kidney stone treatment. Besides, these procedures are still not effective in reducing the high incidence and recurrence rates of kidney stone (Morgan and Pearle, 2016; Zisman, 2017). Therefore, the study of stone formation mechanisms is particularly important in the field of urology. During the last decade, the study of stone mechanisms has gradually increased worldwide. Key areas focus on apoptosis, oxidative stress and crystal adhesion.

Autophagy is a highly conserved and tightly regulated cellular event that allows autophagosomes to isolate organelles, bind to lysosomes, remove damaged organelles or long-lived proteins from the cell, and obtain recycled material for recycling and maintaining cellular homeostasis (Rautou et al., 2010; Choi et al., 2013; Martinet et al., 2009). Autophagy has been shown to be a relevant factor in many diseases (Jacob et al., 2017). Under conditions such as cell starvation and hypoxia, autophagy is often thought to mitigate cellular damage and allow for continued survival (Levine and Klionsky, 2004). Many related studies on autophagy in kidney have shown that enhanced autophagy in the kidney is protective against certain injuries, such as hyperuricemia, ischemia, cisplatin-induced injury and mitochondrial metabolic stress, etc. (Isaka et al., 2015; Kimura et al., 2011; Takahashi et al., 2012; Kimura et al., 2013; Namba et al., 2014; Maejima et al., 2013). However, many studies have also shown that activation of autophagy through certain pathways can exacerbate cellular damage. Excessive activation of autophagy has been shown to be detrimental under a certain degree of pressure and may further damage intracellular substances and structures, especially mitochondria and endoplasmic reticulum. In addition, excessive autophagy exacerbates cellular oxidative stress and even induces cell death (Debnath et al., 2005; Law et al., 2010). Furthermore, we have noted that autophagy is a double-edged sword for apoptosis (Eisenberg-Lerner et al., 2009; Nikoletopoulou et al., 2013). This dual relationship between promotion and inhibition depends on the degree of cellular damage or the level of ROS in the cell (Ureshino et al., 2014; Eisenberg-Lerner et al., 2009; Nikoletopoulou et al., 2013), and the point of crosstalk between autophagy and apoptosis is also the focus of future research. These findings suggest that autophagy is a complex cellular event and that autophagy-related events exist in multiple organelles. As a cellular mechanism to remove its own harmful substances, several related studies have shown that the regulation of autophagy may have profound implications for the formation of renal calculi, and this paper presents a systematic review of the literature.

## Materials and methods

This systematic literature review was conducted in accordance with the Preferred Reporting Items for Systematic Reviews and Meta-Analyses (PRISMA) guidelines.

### Search strategy

We searched PubMed, MEDLINE, Embase, and Scopus databases using the MeSH key terms, “autophagy” OR “cellular autophagy”, OR “mitophagy”, AND “urolithiasis”, OR “calcium oxalate”, OR “kidney stone”. Article type, publication date, language, or species were not restricted during the initial search.

### Eligibility criteria

This article includes articles on the relationship between hyperoxaluria, kidney stone and cellular autophagy, and on the pathogenesis of kidney stone concerning autophagy. In order to improve the direct correlation between the included studies and the subjects of this study, the following studies were excluded: Studies on autophagy in diseases of other organs of the urinary system and renal amorphous precipitation injury. In addition, we eliminated articles from the same research institution or the same author with high similarity, as well as articles with retracted manuscripts. This paper focuses more on experimental research and original articles in order to better find clear conditions and conclusions from the original experiments.

### Data collection and description

Two authors (Hao Li and Yingjian Zhou) independently reviewed the titles and abstracts of the articles identified in the initial search on 20 September 2021. Data were extracted from articles that met the eligibility criteria and reassessed in full-text articles during primary and secondary screening, respectively. The differences and discrepancies between the two screenings were resolved through discussion and consensus with other authors. The following data were also extracted from all eligible full-text articles: title, first author, journal name, year, method, type of experimental subject source, and major findings regarding the role of autophagy in renal calculi.

## Results

**FIGURE 1:**
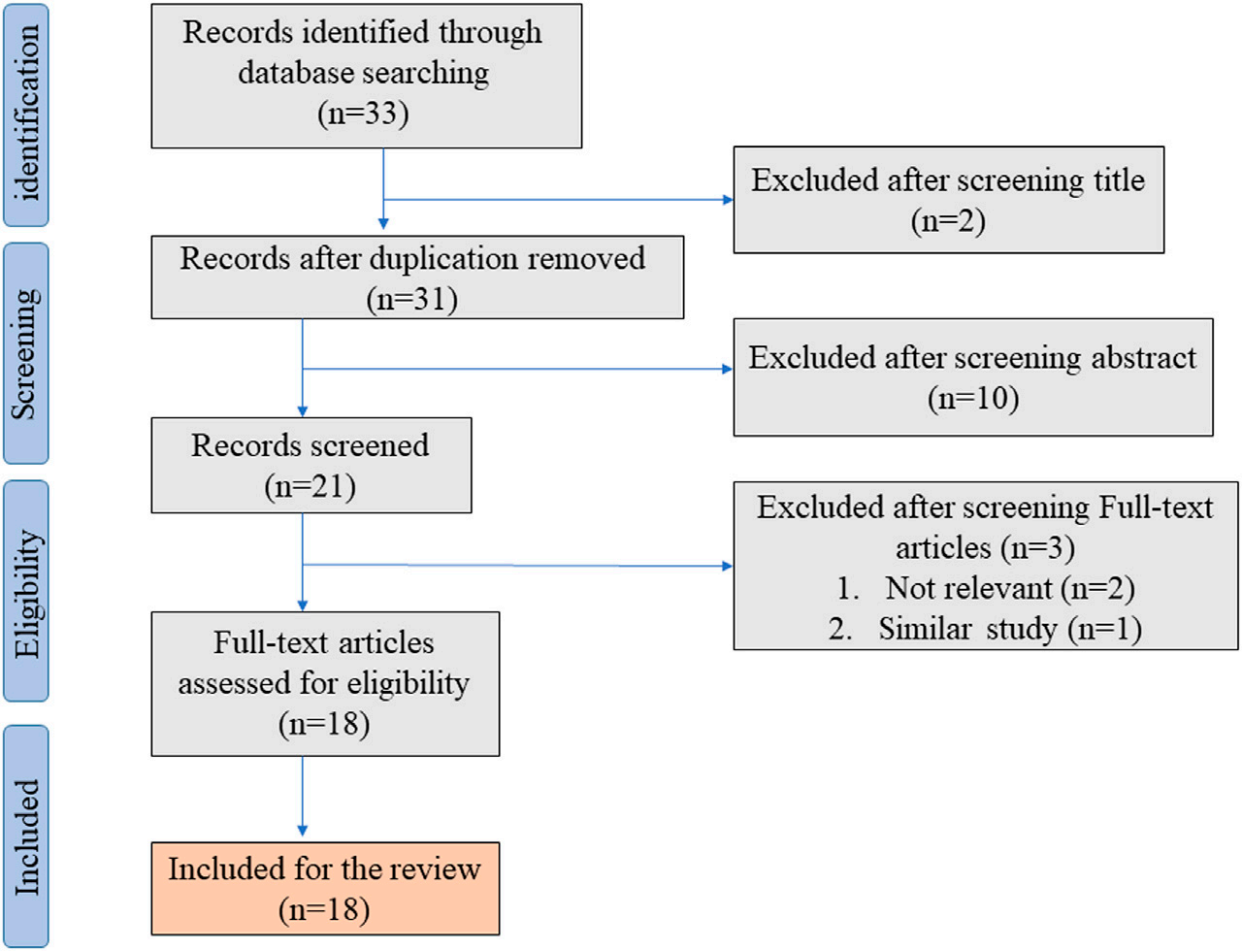
Shows the PRISMA flowchart.

In the initial literature search, we found 33 studies that met the search strategies and criteria in the database. After reviewing the title and abstract, they are finally filtered into 18 articles that meet the standards through full-text reading. Figure 1 shows the PRISMA flowchart. Table 1 summarizes 18 articles. These articles are mainly from China and Japan and include *in vivo* experiments, *in vitro* experiments, and human tissue analysis. Table 1 also contains the titles, journals, years, first authors, major drugs, experimental subjects and major findings of these articles.

**TABLE 1:** Summary table of literature for systematic review.

Tittle	First Author	Year	Journal	In vitro	Key Related Drugs	In vivo	Key Related Drugs	Human tissues	Main finding in autophagy and kidney stone
Total flavone of Desmodium styracifolium relieved apoptosis and autophagy of COM-induced HK-2 cells by regulating KIM-1 via p38/MAPK pathway	Xie H	2017	Molecular and Cellular Biochemistry	HK-2 cells	COM(2mM ) ; TFDS (50 μg/mL), SB203580(50 μM SB203580)	/	/	/	TFDS inhibited HK-2 cell apoptosis and autophagy by downregulating the p38/MAPK/KIM-1 pathway in a COM co-incubation environment.
Inhibition of autophagy-attenuated calcium oxalate crystal-induced renal tubular epithelial cell injury in vivo and in vitro	Liu Y	2017	Oncotarget	HK-2 cells	CaOx crystals (4 mM) ; Rapamycin((10 µM) , 3-MA (5 mM), NAC(5 mM) , CAT (2000 U/ml)	/	/	28 kidney tissue specimens from patients with calcium oxalate kidney stones; 12 normal kidney tissue samples from patients who underwent radical nephrectomy of tumors	Autophagy induced by CaOx crystals via the ROS pathway exacerbates renal tubular epithelial cell injury.
Inhibition of Autophagy Attenuated Ethylene Glycol Induced Crystals Deposition and Renal Injury in a Rat Model of Nephrolithiasis	Liu Y	2018	Kidney and Blood Pressure Research	/	/	Sprague-Dawley rats	EG(0.75%) ; Rapamycin (0.25 mg/kg/d), Chloroquine (60 mg/kg/d)	/	Inhibition of autophagy alleviates EG-induced crystal deposition and kidney injury in a rat model of renal calculi.
Autophagy inhibition attenuates hyperoxaluria-induced renal tubular oxidative injury and calcium oxalate crystal depositions in the rat kidney	Duan X	2018	Redox Biology	NRK-52E cells	Oxalate(0.75 mM) ; Rapamycin (5μM) , Chloroquine (5μM)	Sprague-Dawley rats	EG (1%) ; Chloroquine (30 mg/kg/d), Rapamycin (0.25 mg/kg/d)	/	Autophagy inhibition attenuates oxalic acid-induced oxidative damage in renal tubular cells and CaOx crystal deposition in rat kidney, at least in part by inhibiting activation of the p38 signaling pathway.
Ethyl Pyruvate Attenuates CaCl2-Induced Tubular Epithelial Cell Injury by Inhibiting Autophagy and Inflammatory Responses	Zhao J	2018	Kidney and Blood Pressure Research	HK-2 cells	CaCl2(1.0 mg/ml ) ; EP (2.5 mM )	/	/	/	Under CaCl2 conditions, EP attenuated the level of autophagy, and thus cell damage, probably by interfering with HMGB1 released outside HK-2 cells and inhibiting the competitive binding between HMGB1 and Beclin-1 and Bcl-2.
Taurine attenuates OTA-promoted PCV2 replication through blocking ROS-dependent autophagy via inhibiting AMPK/mTOR signaling pathway	Zhai N	2018	Chemico-Biological Interactions	HK-2 cells	CaOx crystals (4mmol/L) ; Tau (150 μmol/L)	Sprague-Dawley rats	EG(0.75%) Tau (300 mg/kg/d) , MK2206 (90 mg/kg/48h)	/	Taurine inhibits ROS-dependent autophagy through activation of CaOx crystal-induced Akt/mTOR signaling pathway in HK-2 cells and kidney injury, thereby exerting a cytoprotective effect.
Calcifying nanoparticles induce cytotoxicity mediated by ROS-JNK signaling pathways	Wu J	2018	Urolithiasis	HK-2 cells	CNP (2 MCF)	/	/	/	CNPs are phagocytosed by HK-2 cells, leading to autophagy, apoptosis, and ROS production, in part by activating the JNK signaling pathway. the ROS and JNK pathways may be involved in CNP-induced cell injury and kidney stone formation.
Exosomes from miR‐20b‐3p‐overexpressing stromal cells ameliorate calcium oxalate deposition in rat kidney	Shi J	2019	Journal of Cellular and Molecular Medicine	NRK‐52E cells ; ADSCs	Oxalate(0.75 mmol/L) , miR‐20b‐3p mimics(20 nmol/L ); miR‐20b‐3p‐enriched exosomes , TLR4 overexpression vector , ATG7 overexpression vector	Sprague-Dawley rats	EG(1%) ; miR‐20b‐3p‐enriched exosomes (400 μg of protein)	30 urine specimens from patients with kidney stones; 30 urine specimens from normal subjects	Co-culture with mir-20b-3p-enriched exosomes can be used to prevent kidney stones by inhibiting ATG7 and tlr4 to attenuate oxalate-induced cellular autophagy and inflammatory responses.
Curcumin ameliorates glyoxylate-induced calcium oxalate deposition and renal injuries in mice	Li Y	2019	Phytomedicine	/	/	C57BL/6 mice	GOX (100 mg/kg/d) ; curcumin(50 or 100 mg/kg/d)	/	In a mouse kidney stone model, curcumin inhibited autophagy, oxidative stress and thus attenuated kidney injury, which was associated with the Nrf2 pathway.
Autophagy-endoplasmic reticulum stress inhibition mechanism of superoxide dismutase in the formation of calcium oxalate kidney stones	Kang J	2020	Biomedicine & Pharmacotherapy	/	/	Sprague-Dawley rats	EG (0.75% ) ; ATO(2 mg/kg/d), DETC (350 mg/kg/48h)	/	Enhanced SOD activity protects the kidney by reducing the autophagy-ERS response and CaOx kidney stone formation, which may be related to the PI3K/AKT pathway.
Deregulated MTOR (mechanistic target of rapamycin kinase) is responsible for autophagy defects exacerbating kidney stone development	Unno R	2020	AUTOPHAGY	M-1 cells	COM(20 μg/cm2), Torin1(1 μM), 3-MA (5mM)	GFP-MAP1LC3 transgenic mice and C57BL/6J mice	GOX (80 mg/kg/d) ; Rapamycin (1 mg/kg/d)	Mucosa of renal papillae with plaques and normal papillae without plaques from 23 patients (aged 20-80 years) with idiopathic CaOx stones	Defective autophagy caused by the dysregulated MTOR-TFEB axis may be a potent target for kidney stone formation, and MTOR inhibition may be a prospective approach for the treatment of kidney stones.
LC3 lipidation is essential for TFEB activation during the lysosomal damage response to kidney injury	Nakamura S	2020	Nature Cell Biology	PTECs	CaOX(100μg/ml)	Conditional PTEC-specific Atg5-deficient mice on a C57BL/6 background	sodium oxalate (75mg/kg )	Kidney tissue extracted from patients with crystalline nephropathy (acute phosphate nephropathy, CaOx nephropathy and monoclonal light chain renal tubulopathy)	TFEB activation slows cellular damage by promoting lysosomal repair in a calcium oxalate kidney stone model. Decreased TFEB nuclear translocation in human crystalline nephropathy.
Effect of endoplasmic reticulum stress-mediated excessive autophagy on apoptosis and formation of kidney stones	Sun Y	2020	Life Sciences	/	/	Sprague–Dawley rats	EG(1%) ; 4-PBA (100 mg/kg/d), Chloroquine (60mg/kg/d)	/	ERS induces excessive autophagy through the PERK-eIF2α pathway, which induces cell damage and apoptosis.
miR-155 facilitates calcium oxalate crystal-induced HK-2 cell injury via targeting PI3K associated autophagy	Chen X	2020	Experimental and Molecular Pathology	HK-2 cells	CaOx crystals ( 2 mM) ; Rapamycin (10 μM) , 3-MA ( 5 mM)	/	/	Clinical kidney tissue from patients ; Peripheral blood samples from 20 patients diagnosed with calcium oxalate kidney stones and 20 normal volunteers	miR-155 promotes CaOx crystal-induced renal tubular epithelial cell injury via PI3K/Akt/mTOR-mediated autophagy
Oxalate Activates Autophagy to Induce Ferroptosis of Renal Tubular Epithelial Cells and Participates in the Formation of Kidney Stones	Song Q	2021	Oxidative Medicine and Cellular Longevity	HK-2 cells	oxalate(2mmol/L) ; BECN1 over expression , NCOA4 knockdown	Sprague-Dawley rats	EG(1%)	/	The effect of oxalic acid on Ferroptosis in HK-2 cells was caused by autophagy activation, and knockdown of NCOA4 ameliorated this effect.
Resveratrol Attenuates Oxalate-Induced Renal Oxidative Injury and Calcium Oxalate Crystal Deposition by Regulating TFEB-Induced Autophagy Pathway	Wu Y	2021	Frontiers in Cell and Developmental Biology	NRK-52E cells	Oxalate (800µmol/L) ; RSV (800µmol/L)	Sprague-Dawley rats	GAM (100 mg/kg/d) ; RSV (10 mg/kg/d)	/	RSV exerts its antioxidant stress activity and preventive effects to prevent kidney stone formation, at least in part by activating TFEB-induced autophagy .
Exosomes derived from calcium oxalate-treated macrophages promote apoptosis of HK-2 cells by promoting autophagy	Yan L	2022	Bioengineered	HK-2cells, THP-1Mø	CaOx crystals (1 mg/mL) ; CaOx-Exo (30μg), 3-MA (5mM)	/	/	/	CaOX-EXO induces apoptosis of HK-2 by promoting autophagy.
Trimethylamine N-oxide promotes hyperoxaluria-induced calcium oxalate deposition and kidney injury by activating autophagy	Dong F	2022	Free Radical Biology and Medicine	HK-2 cells	CaOx crystals (300 μg/ml) ; TMAO (100 μmol/L)	C57Bl/6 mice	sodium oxalate (50 μmol/g); TMAO (1%)	/	TMAO exacerbates hyperoxaluria-induced renal injury by triggering the PERK/ROS pathway, enhances autophagy, apoptosis and inflammation, and promotes CaOx crystal deposition in renal tubular cells.

### Autophagy induction in kidney stone models *in vitro*

Regulation of autophagy has been found in almost all kidney stone models, which can reflect the importance of autophagy in the formation of kidney stone. *In vitro* experiments focused on the induction of kidney stone models and cellular autophagy by the high concentration of COM, CaCl2, Oxalate, CNP, etc. The treatment objects included HK-2, NRK52E and mice RTCs. Xie et al. (2017) first investigated autophagy in kidney stone models using COM co-incubation with HK-2, which exacerbated the upregulation of the autophagy-associated protein LC3-II and the kidney injury marker KIM-1 with the loss of cellular viability in HK-2 after increasing the concentration or time of COM treatment. Liu et al. (2017) evaluated human specimens of calcium oxalate kidney stone and normal kidney tissue using IHC and Western Blotting. Significantly elevated LC3 and BECN1 and a significant increase in autophagic vesicles in human kidney stone specimens signify the presence of upregulation of autophagy in kidney stone formation. In another study conducted by Liu et al., CaOx (0–4 mm) was used to control the dose and time of HK-2. Consistent with the study of Xie, CaOx induced HK-2 autophagy in a dose and time-dependent manner (Liu et al., 2017; Liu et al., 2018). Similarly, when NRK-52E was treated with oxalate, LC3- II expression was increased and P62 expression was down-regulated, indicating an increase in autophagy in cells (Duan et al., 2018). Exposure to CNPs induced significant upregulation of autophagosome and autolysome contents in HK-2, which was shown to be associated with the activation of ROS-JNK signaling (Wu et al., 2018). Co-incubation of 0–2.0 mg/ml of CaCl2 with HK-2 has also been shown to have a dose- and time-dependent induction of autophagy and has been shown to be associated with upregulation of the HMGB1-RAGE/TLR4-NF-κB axis (Zhao et al., 2018).

Unno et al. (2020), found an upregulation of autophagy at the beginning of co-incubation in mouse RTCs co-incubated with CaOx by measurement of MAP1LC3 protein turnover. And after 6–8 h, the upregulation of autophagy was reversed, which correlated with increased protein levels and phosphorylation of SQSTM1. In addition, lysosomes and autophagosomes were more abundant at 4 h after CaOx co-incubation but almost disappeared 8 h after CaOx co-incubation. Co-incubation with CaOx also induced phosphorylation of RPS6KB1/p70S6K and ULK1 in RTCs and down-regulation of nuclear translocation of autophagy-related protein TFEB, while Lamp1 and UVRAG-DT downstream of TFEB also showed corresponding inhibition. These autophagy changes indicated that CaOx induced the activation of mTOR and the phosphorylation and activity inhibition of the nuclear transcription factor TFEB downstream of mTOR. These all point in one direction -----COM processing impairs autophagy in mouse RTCs, which is different from autophagy activation as previously thought. In addition, in human CaOx nephropathy specimens, damaged mitochondria, lysosomes, and crystals were found in the patch-covered mucosa, whereas only a few autolysosomes could be found. In addition, the tissue was positive for SQSTM1 and its phosphorylated form, again indicating impaired autophagic flux. Similarly, Nakamura et al. (2020) found that nuclear translocation of TFEB appeared impaired in kidney specimens from stone patients. This complements the idea of impaired autophagy in kidney stone pathogenesis by Unno et al. (2020).

### Autophagy induction of *in vivo* kidney stone models

Rat and mouse models of hyperoxaluria are now widely used in the study of renal calculi (Pan et al., 2013). Liu et al. (2018) conducted the first *in vivo* experiments on kidney stone-related autophagy. In the kidneys of SD rat models (4 weeks) reared with 0.75% EG solution, the expression of LC3-II and BECN1 was significantly increased, and the number of autophagic vesicles was observed to increase under TEM, indicating that autophagy was up-regulated in the rat kidney stone formation models. In addition, researchers used glyoxylate (100 mg/kg) for intraperitoneal injection to construct mouse kidney stone models, which are similar to the EG models and observed an increase in Beclin-1 with LC3-II and a decrease in p62 in kidney specimens (Li et al., 2019). This upregulation of autophagy was also confirmed by the increase of Beclin-1, LC3B/A and downregulation of p62 in mouse models of hyperoxaluria constructed with sodium oxalate (50 μmol/g, 15 days) (Yan et al., 2022). Although these kidney stone models are constructed in different ways, they jointly prove that autophagy is up-regulated in the pathogenesis of kidney stone.

However, Unno et al. (2020) found some phenomena that differ from the view of autophagy upregulation in the construction of kidney stone models. In the kidney stone formation models constructed by GFP-MAP1LC3 transgenic mice injected intraperitoneally with glyoxylate (80 mg/kg), autolysosomes containing damaged organelles were found in RTCs during the period of nonnephrocalcinosis (12 h before). The measurement and analysis of MAP1LC3 spots and SQSTM1 showed that autophagosome accumulation occurred at the initial stage after GOX injection (0–6 h). However, after 4 days of GOX intraperitoneal injection, a large number of cytoplasmic vesicles and crystals were observed in RTCs. In addition, co-localization of MAP1LC3 and SQSTEM1 after crystal formation confirmed that autophagy was impaired in RTCs after crystal formation (Unno et al., 2020).

### Effect of autophagy downregulation of *in vitro* kidney stone models

*In vitro* downregulation of autophagy can reduce the damage and adhesion of stones to cells. Xie et al. (2017) treated HK-2 with TFDS and inhibited COM-induced autophagy. In addition, COM-induced apoptosis, crystal adhesion, and the upregulation of p-p38 were reversed. In particular, the inhibition of autophagy and damage was further enhanced when acting in conjunction with p38/MAPK pathway inhibitors, whereas KIM-1 overexpression reversed this protective effect, allowing increased crystal adhesion. These findings suggest that this autophagy and damage is at least partially mediated by the p38/MAPK/KIM-1 pathway. Similarly, chloroquine treatment or knockdown of BECN1 also downregulated autophagy. This downregulation of autophagy was associated with the phosphorylation of p38, which alleviated the loss of cellular viability and oxidative stress associated with COM co-incubation.

Liu et al. (2017) found that 3-MA pretreatment similarly downgraded autophagy in kidney stone models and alleviated CaOx stress-induced apoptosis and loss of cellular viability in HK-2. The study by Zhao et al. (2018) focused on the HMGB1-RAGE/TLR4-NF-κB pathway. EP, an inhibitor of HMGB1, inhibited the activation of HMGB1-RAGE/TLR4-NF-kβ and alleviated the release of inflammatory cytokines and cellular injury in HK-2 models treated with CaCl2. Western Bolting detected the downregulation of Beclin-1 and LC3, as well as observation by confocal microscopy, indicating the presence of autophagy inhibition by EP in HK-2. In addition, EP induced an increase in Bcl-2 levels as well as an upregulation of extracellular HMGB1 levels, suggesting that: the protective effect of EP on HK-2 under the pressure of kidney stone formation may be achieved through the interference of HMGB1 on the binding between Bcl-2 and BECN-1, and the co-regulation of autophagy by downstream signals mediated by the role of HMGB1 in the extracellular receptor.

In the regulation of autophagy in the Akt/mTOR pathway, Tau has been shown to reverse the downregulation of Akt and mTOR phosphorylation in HK-2 under CaOx pressure, thereby inhibiting the increase of autophagy flux (Zhai et al., 2018). Chen et al. (2020) used CaOx co-incubated with HK-2 to construct *in vitro* kidney stone models. In the anti-miR-155 transfection group, dual luciferase reporter assay and mRNA and protein expression studies showed that miR-155 silencing mediated autophagy reduction was accompanied by PI3K/Akt/mTOR axis upregulation. Importantly, the restoration of the Akt/mTOR pathway in both experiments attenuated apoptosis and cellular injury under the stress of kidney stone formation.

ROS is a major marker of oxidative stress. ROS occupies an important position in autophagy induction and degradation. In addition, past studies have shown that autophagy can also modulate ROS levels by removing damaged proteins and organelles. High doses of ROS in cells are considered detrimental and are thought to be upstream inducers in autophagy (Ureshino et al., 2014; Eisenberg-Lerner et al., 2009). The level of ROS has been shown to be the threshold for protective or excessive autophagy (Nikoletopoulou et al., 2013, Moretti et al., 2007). Liu et al. (2017) applied the antioxidants NAC or catalase *in vitro*, both of which inhibited the accumulation of ROS and subsequently excessive autophagy and injury in CaOx-induced HK-2 kidney stone models. In addition, several studies have shown that multiple pathway-mediated downregulations of autophagy *in vitro* all reduced the concentration of ROS, which in turn ameliorates oxidative stress-induced cellular damage (Duan et al., 2018; Zhai et al., 2018; Chen et al., 2020). This indicates that autophagy and ROS are interactional in the formation of kidney stone, and jointly participate in the pathogenesis of hyperoxaluria.

The microenvironment of renal tubular epithelial cells can affect autophagy, among which macrophages and ADSCs are considered to play a significant role. ADSCs-derived miR-20b-3p-enriched exosomes co-cultured with NRK-52E cells were shown to achieve autophagic downregulation through ATG7 inhibition, which attenuated oxalate-induced crystal adhesion and loss of cellular viability (Shi et al., 2019). TMAO is thought to be involved in gut microbial metabolism. Dong et al. (2022) applied TMAO in the HK-2 kidney stone models constructed by CaOx, and TMAO induced a marked up-regulation of autophagy, which was reversed by the silencing of PERK. Similar to the previous pathways, inhibition of autophagy alleviated apoptosis and detected an improvement in oxidative stress, crystal adhesion and cytotoxicity. In recent studies, CaOx was co-incubated with THP-1 macrophages and induced to produce exosomes (CaOx-Exo). Co-incubation of CaOx-ExO with HK-2 cells induced significant up-regulation of autophagy. The application of 3-MA can inhibit the up-regulation of autophagy, and the reduction of autophagy also attenuates the expression of Bax and caspase-3 induced by exosomes (Yan et al., 2022). These two studies confirmed the protective effect of autophagy downregulation on kidney stone formation and also suggested that other factors *in vivo* have regulatory effects on kidney stone formation. Compared with *in vitro* experiments with relatively single conditions, *in vivo* experiments may have more scientific significance. In the following discussion, we will also carry out further analysis through the literature *in vivo*.

However, in the COM-induced M-1 models, Unno et al. (2020)found an experimental phenomenon different from the above, showing the injury effect of autophagy down-regulation in kidney stone. In the adhesion phase of COM-based kidney stone *in vitro*, Unno et al. found time-dependent mitochondrial damage by detecting mitochondrial signaling, TOMMM20 signaling, and Pink expression levels. In addition, further studies by Unno et al. confirmed that SOD1 activity gradually decreased with COM co-culture time, and this promotion of oxidative stress was accompanied by the down-regulation of autophagy. In addition, they found damage to lysosomes. One possible explanation is that the downregulation of autophagy induced by the kidney stone environment prevents the removal of damaged organelles and further induces ROS burst, which is also accompanied by the release of inflammatory factors. The researchers further promoted COM-induced autophagy down-regulation in mice RTCs by administering 3-MA. Interestingly, this inhibition of autophagy induces more severe inflammation, oxidative stress, and crystal adhesion. Similarly, In the study by Wu et al. (2021), 3-MA administration inhibited autophagy and weakened the protective effect of RSV on NRE-52E cells under oxalate stress. In the study of Chen et al. (2020), 3-MA and CaOx were also used for the HK-2 experiment, but the inhibition of autophagy induced the decline of apoptosis. This difference may be related to the experimental materials and induction conditions.

### Effect of autophagy upregulation of *in vitro* kidney stone models

Some studies have shown that up-regulation of autophagy *in vitro* may promote cellular damage and adhesion of kidney stones. In kidney stone experiments by Xie et al. (2017), which investigated the p38/MAPK/KIM-1 pathway, KIM-1 overexpression induced the upregulation of autophagy, which exacerbated the loss of cellular viability of HK-2. Rapamycin further activated autophagy in the NRK-52E kidney stone models constructed by oxalate, and induced more obvious mitochondrial membrane potential loss and crystal adhesion. In addition, p38 was found to be significantly up-regulated in this study (Duan et al., 2018). In the experiment of Chen et al. (2020), co-incubation of HK-2 and CaOx was used instead, and strong inhibition of Akt and mTOR was observed after the Rapa application. Moreover, this significant activation of autophagy reverses the protective effect of miR-155 inhibition.

In the studies of kidney stone in relation to oxidative stress, few experiments have been conducted to regulate autophagy by directly inducing ROS increase. However, a number of experiments have confirmed the dependence of oxidative stress on the concentration and time of environmental mimics for renal stones. Liu et al. (2018) first demonstrated the positive correlation of ROS with CaOx concentration in CaOx-co-incubated HK-2 kidney stone models. The positive correlation between ROS and CNPs treatment time was confirmed in the CNPs-induced HK-2 kidney stone models (Wu et al., 2018). In other studies, up-regulation of autophagy *in vitro* was shown to activate oxidative stress, including down-regulation of mTOR (Duan et al., 2018), overexpression of BECN1 (Song et al., 2021), and phosphorylation of PERK (Dong et al., 2022). In addition, knockdown of BECN1 inhibited oxalate-induced Ferroptosis by suppressing the protein level of GPX4. This correlation of autophagy with Ferroptosis was confirmed in subsequent experiments, and NCOA4 acted as a bridge (Song et al., 2021).

In the study of HK-2 ERS induced by CaOx exposure, Dong et al. found that PERK knockdown inhibited TMAO-induced autophagy enhancement and alleviated TMAO-induced cellular viability loss, oxidative stress and crystal adhesion, which indicated the role of URP and ERS in kidney stone-related autophagy (Dong et al., 2022).

ADSC-secreted miR-20b-3p exosomes have also been studied *in vitro*, and ADSC transplantation has previously been shown to be effective in the treatment of kidney stone (Wang et al., 2018; Teng et al., 2015). In the study by Shi et al. (2019), overexpression of TLR4 or ATG7 reversed miR-20b-3p-induced downregulation of autophagy and miR-20b-3p-mediated cytoprotection. In addition, exosomes (CaOx-Exo) produced by THP-1 macrophages co-incubated with CaOx induced significant up-regulation of autophagy in HK-2 cells, accompanied by a significant increase in apoptosis (Yan et al., 2022).

In contrast, upregulation of TFEB-associated autophagy in kidney stone showed a protective effect on cells *in vitro*. In most of the experiments mentioned above, the mTOR inhibitor rapamycin was used to enhance autophagy, but rapamycin has no significant specificity for mTOR substrates (Kang et al., 2013). In contrast to the previous experiments, Unno et al. used torin1, an autophagy enhancer with strong TFEB specificity (Thoreen et al., 2009). Torin1 was applied to M-1 cells co-incubated with COM, which induced a significant upregulation of autophagy, thereby alleviating lysosomal and mitochondrial damage. The experimental results also showed that this upregulation of autophagy was related to the improvement of inflammatory response and crystal adhesion (Unno et al., 2020). Wu et al. (2021) applied resveratrol in the NRK-52E models constructed by oxalate, which promoted autophagy and reversed the inflammatory response, loss of viability and crystal adhesion under environmental stress of kidney stone. This promotion of autophagy has been shown to be associated with the up-regulation of nuclear translocation of TFEB. All *in vitro* autophagy upregulation with renal calculi protective properties was targeted to TFEB, confirming the importance of TFEB for renal stone treatment. Even as a classical substrate of mTOR, TFEB has also been shown to be regulated through other mTOR-independent pathways (Chen et al., 2021). Direct targeting of TFEB will be critical in subsequent studies.

### Effect of autophagy downregulation of *in vivo* kidney stone models

Partial studies suggest that autophagy downregulation inhibits kidney stone formation in models *in vivo*. Liu et al. (2018) first investigated the *in vivo* autophagy regulation of kidney stone by applying chloroquine to EG-constructed kidney stone models in SD rats. The detection of markers of renal injury, renal function, and renal pathology indicated for the first time the protective effect of autophagy inhibition *in vivo* on kidney stone and the possibility of treating kidney stone. Duan’s experiments (2018), similar to Liu’s, complemented the improvement of oxidative stress and mitochondrial damage in hyperoxaluria kidneys by down-regulated autophagy and determined the dependence of down-regulated autophagy protection on reduced p38 phosphorylation.

In the EG-constructed SD rat models, Tau inhibited EG-induced autophagy upregulation by reversing the down-regulation of the Akt/mTOR pathway and showed improved crystal deposition and apoptosis, as well as oxidative stress (Zhai et al., 2018). ATO was confirmed to effectively increase SOD1 activity and inhibit excessive autophagy in EG-constructed SD rat kidney stone models. In addition, this study also demonstrated renal protection and improved crystal adhesion, which were associated with improved ERS (Kang et al., 2020). Curcumin was shown to reverse the downregulation of Nrf2 and its downstream factors in glyoxylate-constructed mouse kidney stone models. These downstream factors regulate inflammation, oxidative stress and other cellular events, and play a role in the inhibition of autophagy and apoptosis, also in kidney antioxidant protection (Li et al., 2019).

Research on endoplasmic reticulum stress has focused on *in vivo* studies. 4-PBA, a classical inhibitor of endoplasmic reticulum stress, significantly inhibited endoplasmic reticulum stress and attenuated excessive autophagy in kidney stone models constructed in SD rats, exhibiting an improvement in renal histopathology and renal crystal deposition, showing similar efficacy to CQ (Sun et al., 2020).

Based on the low survival rate of ADSC transplants, Shi et al. (2019) put the therapeutic idea of miR-20b-3p enriched exosomes from ADSCs into animal testing. The result of Western Blotting showed that the exosomes acted as an inhibitor of autophagy in EG-induced SD rats and reduced hyperoxaluria, downregulated markers of renal injury, and played a protective role. The above experimental conclusions indicate the protective effect of down-regulated autophagy *in vivo*, but the injurious effect of down-regulated autophagy has also been found in recent studies. In Nakamura’s study (2020), calcium oxalate kidney stone models were constructed using gene-edited mice and sodium oxalate. The impairment of TFEB translocation in LRP2-positive PTECs of PTEC-specific ATG5-deficient mice suggests a dependence of TFEB nuclear translocation on the ATG system. In addition, TFEB-specific knockout mice suffered a greater renal injury and oxidative stress after oxalate exposure and even exhibited higher mortality over time (3/18), whereas no death was found in controls. This suggests that autophagy is essential in the protection of kidney stone *in vivo*. Loss or overregulation of autophagy has been shown to be deleterious in kidney stone. At the same time, TFEB may become a key target for renal stone autophagy research.

### Effect of autophagy upregulation of *in vivo* kidney stone models

Some studies have shown that upregulation of autophagy *in vivo* will promote the formation of kidney stones in the models. Rapa has been applied to autophagy induction *in vivo* many times in the literature included in this article, which is often related to the regulation of mTOR, an important autophagy regulatory protein. In the experiments of Liu et al. (2018), the application of Rapa in the EG-constructed rat models induced further enhanced autophagy, which aggravated mitochondrial damage, renal function and tubular damage, and crystal adhesion. Duan et al. (2018) also used the EG-induced rat kidney stone models and observed that autophagy activation enhanced renal oxidative stress and exacerbated the loss of cellular viability. In addition, they demonstrated that up-regulated autophagy-mediated impairment was associated with p38 activation.

DETC inhibited the activity of SOD in rats, and triggered the aggravation of ERS and the upregulation of autophagy in the ethylene glycol constructed rat models, which further aggravated the kidney injury caused by kidney stone (Kang et al., 2020).

In glyoxylate-induced kidney stone mouse models, the results were quite different. In the study by Unno et al. (2020), administration of rapamycin was confirmed to restore nuclear translocation of TFEB and to suppress GOX-induced increase of SQSTM1 with p-SQSTM1. In addition, abundant autolysosomes were observed under TEM, and these phenomena signify that Rapa successfully induced autophagy upregulation *in vivo*, and this upregulation was shown to improve the inflammatory response and kidney stone formation. In addition, Unno et al. (2020) found that scavengers of ROS also reverse the downregulation of autophagy *in vivo* and played a protective role in kidney stone. Similarly, Wu et al. (2021)performed intragastric administration of RSV in the rat GAM models, which induced upregulation of renal autophagy. And this autophagic upregulation also alleviated CaOx deposition and renal injury and improved oxidative stress induced by the renal stone environment *in vivo*.

## Discussion

The discussion in this systematic review focuses on the function of autophagy in kidney stone. It is apparent that more regulation pathways focus on the inhibition of autophagy, and many studies suggest that kidney stone-related autophagy is excessive autophagy that is detrimental to the cells. Most of the autophagy upregulation in subsequent experiments also indicated that it would cause more serious oxidative stress, damage and apoptosis. There are many crosstalk points between autophagy and apoptosis, which also makes it possible for apoptosis and autophagy to interact with each other (Kyriakis et al., 1994; Eisenberg-Lerner et al., 2009). In addition, previous studies have shown that autophagy accompanies rather than causes cellular death in many cellular contexts (Kroemer and Levine, 2008). The protective and apoptotic effects of autophagy are unified. Protective autophagy eliminates damaged organelles, and mitigates oxidative stress and cellular damage, thus restoring cellular vitality. In contrast, excessive autophagy may induce apoptosis promotion, which directly leads to cellular death. The promotion of autophagy may induce the enhancement of protective autophagy, and may also induce excessive autophagy that promotes apoptosis. We need to further investigate the relationship between apoptosis and autophagy in renal calculi through experiments, and the key point is to study the crosstalk points and the boundary between them.

Critically, we found in our discussion of the TFEB pathway that almost all protective autophagy is associated with TFEB. Although mTOR is regarded as an important regulator of TFEB, we have noted other regulatory pathways of TFEB, as well as the diversity of mTOR substrates. (Kang et al., 2013; Thoreen et al., 2009). Furthermore, the regulation of mTOR does not necessarily activate only the autophagic pathway, and the regulation of mTOR on other cellular events in kidney stone models has not been further explored. In the literature screened by the authors, it was found that one drug may lead to multiple regulatory pathways. In addition, autophagy is often associated with other cellular events, such as inflammatory factor release, ERS, and oxidative stress. These are related to cellular damage. Among some of the literature included in this paper, drug regulation induces the regulation of more than two cellular events simultaneously, one of which is autophagy and the other regulates cellular events such as inflammation or oxidative stress through other transduction pathways. This may then interfere with a separate discussion of the role of autophagy in kidney stone. Highly specialized modulators of intracellular substances, such as siRNA and shRNA, as well as high-targeting autophagy modulators such as torin1, are significant in future studies.

The only signaling pathway identified so far that induces protective autophagy is the TFEB pathway. We hypothesized that autophagy in kidney stone is excessive autophagy as concluded by many studies, whereas further nuclear translocation of TFEB promotes autophagy but induces cytoprotective properties, which is contradictory to the idea of excessive autophagy. This may imply that the injury induced by using drugs to up-regulate autophagy in the kidney stone models may be only a phenotype. This damage may be mediated by other cellular events other than autophagy alone. Another explanation is that enhanced autophagy does not achieve an obvious repair effect, and at the same time, the damage caused by other cellular events induced by drug regulation in the experimental setting is stronger, so the repair function of autophagy is obscured. And these ideas remain only conjectures and need to be confirmed by experiments that further enhance autophagy and remove interference from other cellular events.

In addition, past literature has demonstrated that the boundary between excessive and protective autophagy depends on ROS levels (Nikoletopoulou et al., 2013; Moretti et al., 2007; Tzou et al., 2016). Then, whether the conditions used for models induction of kidney stones are reasonable for simulating the pathophysiological processes of real patients needs further confirmation. In the real situation, the cellular pressure is relatively low, so the up-regulation of autophagy may be protective autophagy, but in the experimental environment of high cellular pressure, the autophagy is harmful and excessive. Real human CaOx nephropathy develops over a long period of time, and reports suggest that the recurrence of CaOx nephropathy tends to occur after several years (Rule et al., 2014). Yet the experiments we discuss are often scaled down to 4 weeks, so the pressure must be multiplied several times in both cellular and animal models of kidney stone. In addition, past studies have shown that Oxalate promotes epithelial cell proliferation at lower concentrations, suggesting that dosage is a point of concern (Kohjimoto et al., 1996). The inducer concentration derived in the pre-experiments, when there was a significant decrease in cellular viability with the application of the inducer, was applied to the subsequent experiments, which necessarily differed from the concentration and resulting cellular effects in the real pathophysiological process. This concentration may exceed the limit of protective autophagy, which induces excessive autophagy and leads to cellular damage. However, in the case of down-regulating the concentration of the reagent for inducing kidney stone, whether the up-regulation of autophagy still plays a damaging role in kidney stone is unsure, which require us to lower the dosage, extend the experimental time, and ensure the efficiency and practicality of the experiment at the same time. This situation has not been considered and experimented with in previous studies, which needs to be confirmed by further experiments.

The authors believe that inhibition of autophagy may not be an appropriate treatment. After inhibition of autophagy, cellular damage decreased for a short time. However, since autophagy is an important cell repair mechanism, cellular damage cannot be repaired timely. Over time, cellular damage accumulates to a certain level and eventually goes to apoptosis, thus autophagy inhibition may be more of a delay than a cure for renal stone recurrence. Moreover, after reading all the relevant literature in the past, we did not observe enhanced autophagy in kidney stone models *in vitro* and *in vivo* that allows renal function indicators to return to the normal level, suggesting that our idea is reasonable. In addition, whether the damage caused by kidney stone to other kidney cells after downregulation of autophagy is repaired and removed *in vivo* has not been studied thoroughly. Since kidney stone treatment is a long-term process, it is also important to consider whether long-term autophagy inhibition or promotion is beneficial to the body. This requires us to extend the observation period and focus on *in vivo* experiments. Enhanced autophagy, in some studies, also plays a protective role in the pathogenesis of kidney stone, and more beneficially, cells may be able to fundamentally repair cellular damage through autophagy. In addition, literature has shown that the enhancement of autophagy can also inhibit apoptosis in some cases (Ding et al., 2007; Kourou et al., 2007; Castine et al., 2005), making autophagy-enhanced therapies more possible.

In some of the included studies, the use of a single kind of drug may simultaneously induce protection against kidney stone by inhibiting autophagy and retarding oxidative stress. Previous studies have shown that the protectiveness and destructiveness of autophagy are related to ROS content (Moretti et al., 2007; Nikoletopoulou et al., 2013; Tzou et al., 2016). The author proposed the idea that we can select a single drug or a combination of drugs that could alleviate oxidative stress and cellular stress at the same time, and then enhance autophagy to induce enhancement of protective autophagy, so as to remove cellular damage further. Kidney stone has a great negative impact on the life quality of patients and social medical security. If we can solve this problem through the regulation of autophagy, the pressure on both will be alleviated. In our next experiment, we will focus on Fisetin, a drug that may reduce oxidative stress and enhance TFEB-related autophagy.

## Conclusion

This paper systematically reviews the literature on kidney stone-related autophagy including studies on *in vivo*, *in vitro* and human specimens from 2017 to now. Initial studies focused on the therapeutic possibilities of inhibiting autophagy for kidney stone. But in recent *in vitro* and *in vivo* studies, enhanced autophagy has been shown to have a protective effect on kidney stone. In addition, a few pieces of literature have studied human tissue specimens. Interestingly, there is a controversy in different literature as to whether autophagy is upregulated or downregulated in calcium oxalate kidney stone specimens. This may be related to the site of tissue extraction. Autophagy is a classical cellular self-repair mechanism. Although the inhibition of autophagy expresses the reduction of cellular damage in a short time, cellular damage cannot be well repaired by autophagy with the accumulation of damage. And the accumulation of cellular damage will eventually lead to apoptosis. However, the up-regulation of autophagy can better eliminate cellular damage and prevent the final apoptosis. In addition, previous studies have shown that the occurrence of protective and damaging autophagy depends on ROS levels (Moretti et al., 2007; Nikoletopoulou et al., 2013; Tzou et al., 2016). Therefore, the authors proposed the idea for future experiments: the combination of drugs downregulates oxidative stress and enhances autophagy to induce protective autophagy and clear cellular damage. This therapy is expected to be effective in treating calcium oxalate kidney stone. In addition, TFEB-targeted agonists should be an important candidate agent.

### Limitations

This systematic review may have several limitations. First, the construction of calcium oxalate kidney stone models varies in many studies, not only in the strains of experimental animals and cells used, but also in the agents used to induce kidney stone, and this inconsistency may affect the validity of the article. Second, due to the pathological differences between crystal deposition in the renal tubules and CaOx stone formation, the results of the mouse kidney stone models may not be applicable to the treatment of kidney stone in humans. In addition, only a few studies have been performed on human specimens, and there is controversy regarding the autophagy changes in human kidney stone specimens.

## Data Availability

The original contributions presented in the study are included in the article/supplementary material, further inquiries can be directed to the corresponding author.

## Glossary

IHC: immunocytochemistry
CaOx: calcium oxalate
COM: calcium oxalate monohydrate
JNK: c-Jun N-terminal kinase
EG: ethylene glycol
MAPK: mitogen-activated protein kinase
Nrf2: nuclear factor erythroid 2-related factor 2
PRISMA: Preferred Reporting Items for Systematic Reviews and Meta-Analyses
ROS: reactive oxygen species
TLR4: toll-like receptor 4
p38: p38 mitogen-activated protein kinase
HMGB1: high mobility group box-1 protein
PERK: Protein kinase R-like endoplasmic reticulum kinase
Akt: protein kinase B
HK-2: human proximal tubule epithelial cell
M-1: murine collecting duct
THP-1: human monocytes
RTC: renal tubular epithelial cell
NRK-52E: a kind of rat renal tubular epithelial cell
RAGE: receptor for advanced glycation endproducts
LC3: Microtubule-Associated Protein 1 Light Chain 3
BECN1: Beclin-1
KIM-1: Kidney injury molecule 1
CNPs: Calcifying nanoparticles
mTOR: mammalian target of rapamycin
p62/SQSTM1: Sequestosome-1
SD rats: Sprague-Dawley rats
GOX: glyoxylate
3-MA: 3-methyladenine
MAP1LC3B: microtubule-associated protein 1 light chain 3 beta
NAC: N-acetyl-l-cysteine
TFEB: transcription factor EB
TEM: transmission electron microscopy
Rapa: Rapamycin
NCOA4: Nuclear receptor coactivator 4
TMAO: Trimethylamine N-oxide
ADSC: adipose‐derived stromal cell
ATG: autophagy‐related gene
Bax: BCL2-Associated X
Bcl-2: B-cell lymphoma-2
TFDS: Total flavone of *Desmodium* styracifolium
Tau: Taurine
SOD: Superoxide dismutase
ATO: Atorvastatin
DETC: Diethyldithiocarbamic acid
RSV: Resveratrol
GAM: glyoxylic acid monohydrate
4-PBA: 4-Phenylbutyric acid
UPR: Unfolded protein response
ERS: endoplasmic reticulum stress
PTEC: immortalized kidney proximal tubular epithelial cells
